# Virulence Differences among *Melissococcus plutonius* Strains with Different Genetic Backgrounds in *Apis mellifera* Larvae under an Improved Experimental Condition

**DOI:** 10.1038/srep33329

**Published:** 2016-09-14

**Authors:** Keiko Nakamura, Yuko Yamazaki, Akiyo Shiraishi, Sota Kobayashi, Mariko Harada, Mikio Yoshiyama, Makoto Osaki, Masatoshi Okura, Daisuke Takamatsu

**Affiliations:** 1 Research and Business Promotion Division, Research Institute for Animal Science in Biochemistry and Toxicology, Sagamihara, Kanagawa 252-0132, Japan; 2 Division of Viral Diseases and Epidemiology, National Institute of Animal Health, National Agriculture and Food Research Organization, Tsukuba, Ibaraki 305-0856, Japan; 3 Division of Animal Breeding and Reproduction Research, Institute of Livestock and Grassland Science, National Agriculture and Food Research Organization, Tsukuba, Ibaraki 305-0901, Japan; 4 Division of Bacterial and Parasitic Diseases, National Institute of Animal Health, National Agriculture and Food Research Organization, Tsukuba, Ibaraki 305-0856, Japan; 5 The United Graduate School of Veterinary Sciences, Gifu University, Gifu, Gifu 501-1193, Japan

## Abstract

European foulbrood (EFB) caused by *Melissococcus plutonius* is an important bacterial disease of honeybee larvae. *M. plutonius* strains can be grouped into three genetically distinct groups (CC3, CC12 and CC13). Because EFB could not be reproduced in artificially reared honeybee larvae by fastidious strains of CC3 and CC13 previously, we investigated a method to improve experimental conditions using a CC3 strain and found that infection with a potassium-rich diet enhanced proliferation of the fastidious strain in larvae at the early stage of infection, leading to the appearance of clear clinical symptoms. Further comparison of *M. plutonius* virulence under the conditions revealed that the representative strain of CC12 was extremely virulent and killed all tested bees before pupation, whereas the CC3 strain was less virulent than the CC12 strain, and a part of the infected larvae pupated. In contrast, the tested CC13 strain was avirulent, and as with the non-infected control group, most of the infected brood became adult bees, suggesting differences in the insect-level virulence among *M. plutonius* strains with different genetic backgrounds. These strains and the improved experimental infection method to evaluate their virulence will be useful tools for further elucidation of the pathogenic mechanisms of EFB.

European foulbrood (EFB) is an important bacterial disease in honeybee larvae; it has spread globally and is recognised as an economically important disease for apiculture. Using the multilocus sequence typing (MLST) scheme, isolates of the causative agent, *Melissococcus plutonius*, have been assigned to 34 sequence types (STs), and the STs have further been grouped into three genetically distinct groups [clonal complex (CC) 3, CC12 and CC13]^1^,^2^,^3^ (http://pubmlst.org/mplutonius/). According to testing, strains belonging to CC3 and CC13, which were referred to as typical *M. plutonius* in previous studies^3^,^4^,^5^, were fastidious in their culture requirements and could not grow on agar media under Na > K conditions. Therefore, the addition of potassium salt to the culture media is required for the normal growth of these strains^4^. In contrast, strains belonging to CC12, which were referred to as atypical *M. plutonius* in previous studies^3^,^4^,^5^, were not fastidious and grew on agar media even under Na > K conditions^4^.

The pathogenic mechanisms of EFB remain enigmatic. For a comprehensive understanding of EFB, experimental infection of honeybee larvae with well-characterised strains is necessary; however, most of *M. plutonius* strains used for previous experimental infections were not genotyped to the CCs, and the experimental results using such uncharacterised *M. plutonius* strains vary among reports^6^,^7^,^8^,^9^. For example, Bailey failed to produce EFB in honeybee colonies with pure cultures of *M. plutonius* in a previous study^6^. In a report by McKee *et al.*^7^, EFB did not develop in artificially reared larvae by subcultured *M. plutonius*. On the other hand, artificially reared larvae fed with subcultured *M. plutonius* died from EFB in a report by Giersch *et al.*^8^, although a high dose of *M. plutonius* or the combination of *M. plutonius* and *Paenibacillus alvei* was necessary to achieve high mortality in the larvae. Vásquez *et al.*^9^ observed a high mortality of larvae infected with *M. plutonius* strain MP3, the CC of which is also unknown, at 21 days postinfection (pi). Differences in honeybee race/strains, rearing conditions of larvae, inoculum doses, and observation period may be factors causing the different results. In addition, differences in the virulence among the *M. plutonius* strains may also influence the results. Indeed, a large-scale epidemiological study conducted by Budge *et al.* suggested that *M. plutonius* from different CCs might differ in their virulence at both brood frame and colony levels^2^. However, at an individual insect level, it is unknown whether *M. plutonius* strains with different genetic backgrounds differ in the virulence.

In a previous study by Arai *et al.*^4^, artificially reared honeybee (*Apis mellifera*) larvae successfully developed EFB by feeding with subcultured atypical (CC12) strains, while under the same conditions, subcultured typical (CC3 and CC13) strains did not reproduce clear clinical symptoms in larvae, at least during the test period (five days). Because it was suggested that *M. plutonius* loses its virulence when subcultured *in vitro*^6^, the authors presumed at the time that the failure to reproduce EFB by the typical strains might be due to a decline in virulence during the repeated subculture^4^. However, strains belonging to CC3 and CC13 are fastidious, so we cannot rule out the possibility that some of the experimental conditions in the previous study were unsuitable for evaluation of the virulence of such fastidious strains.

Methods to rear honeybee larvae *in vitro* (i.e., in the laboratory) were originally introduced into the field of bee research to analyse honeybee physiology and caste development. The methods have been repeatedly improved, and the *in vitro* rearing technique is being used as a routine method for many applications at present^10^. Standard methods for honeybee larval toxicity tests using artificially reared *A. mellifera* larvae^11^,^12^ have recently appeared on the OECD website (http://www.oecd.org/chemicalsafety/testing/). In this study, on the basis of the rearing methods of larvae described in the OECD guideline and draft guidance^11^,^12^, we improved the experimental infection methods for better evaluation of the virulence of a fastidious *M. plutonius* strain of CC3. Under the improved conditions, we then compared the effect of the infection on larvae among *M. plutonius* strains representing each CC.

## Results and Discussion

### Experimental infection I: Evaluation of the virulence of DAT606 of CC3 [DAT606(CC3)] under conditions similar to those of the previous study

At the first onset, we evaluated the virulence of *M. plutonius* DAT606(CC3) against *A. mellifera* larvae under the experimental condition nos 1 and 2 (Table 1). DAT606(CC3) is a fastidious strain of ST3, which belongs to CC3. The condition nos 1 and 2 were similar to those reported by Arai *et al.*^4^. *M. plutonius* suspension in saline was mixed with an equal volume of Diet A. In the infected group, less than 24-h-old larvae were fed with 10 μl of the mixture (i.e., Day 0 diet), whereas control larvae were fed with 10-μl Diet A diluted with the same volume of saline (Table 1). After 24 h of the peroral infection, all the larvae were fed with a fresh diet by *ad libitum* feeding. However, unlike the previous conditions, in which larvae were transferred to new wells at 24 h pi^4^, larvae in this study were reared in the same wells during the experiments to avoid mechanical damage by grafting. The larvae were monitored for six days, that is, the observation period was one day longer than the previous study^4^.

In the previous study^4^, DAT606(CC3)-infected larvae did not show clinical signs of EFB. The survival rate of the DAT606(CC3)-infected group at day 5 pi (94.3%) was comparable to that of the control group (91.4%). Although larval weight was not recorded in the study, all surviving larvae were well grown. However, larvae of the infected group were indeed infected, and 5.88 × 10^6^ to 1.65 × 10^8^ CFU/larva (6.769 to 8.217 log_10_CFU/larva) of *M. plutonius* were isolated from the larvae^4^.

**Figure 1:**
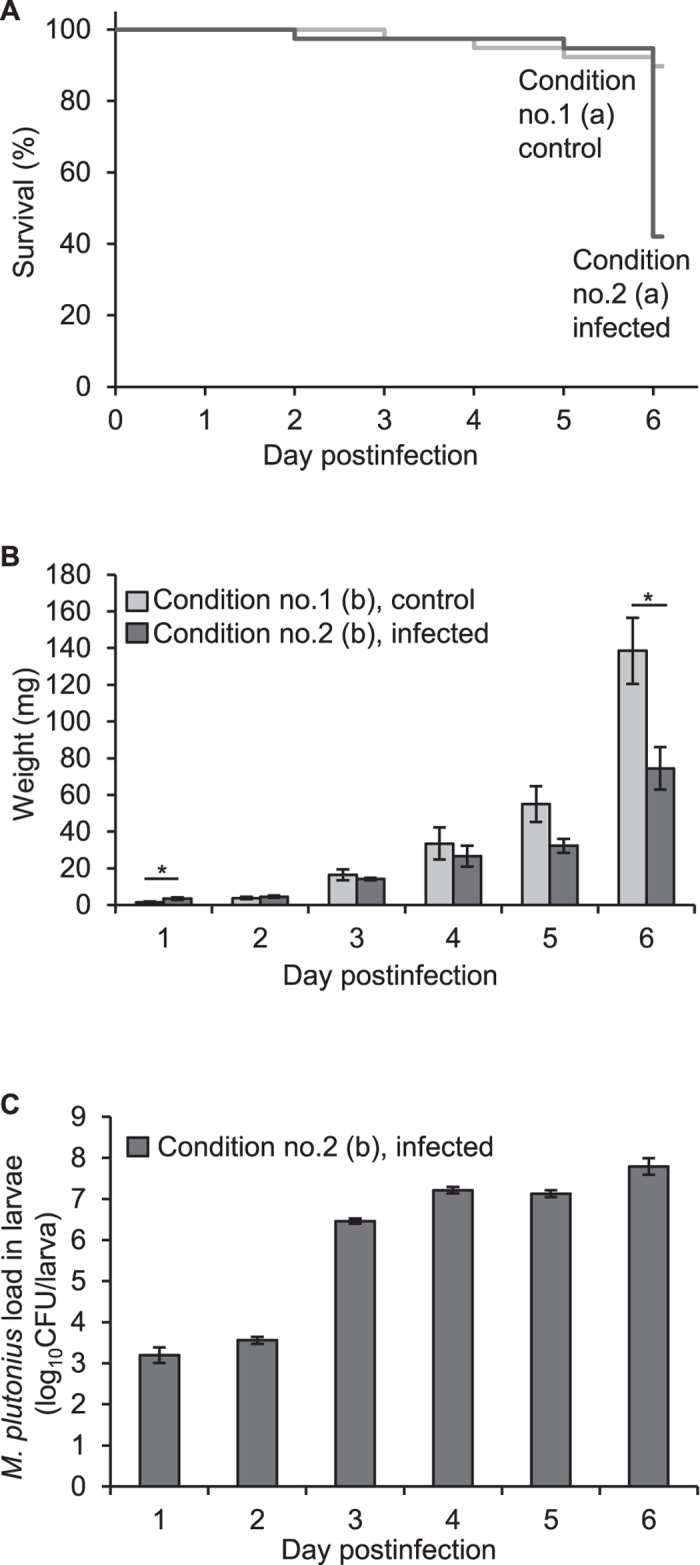
Virulence of *M. plutonius* strain DAT606(CC3) under the previously reported conditions (experimental condition nos 1 and 2). (**A**) Survival of control and infected larvae. Larvae in the infected group (no. 2) were fed with 10-μl Day 0 diet containing *M. plutonius* DAT606(CC3) at a final concentration of 1.7 × 10^7^ CFU/ml (Supplementary Table S1). Survival rates of the control group at days 5 and 6 pi were 92.3% and 89.7%, respectively. Survival rates of the DAT606(CC3)-infected group at days 5 and 6 pi were 94.7% and 42.1%, respectively. (**B**) Larval weight presented as mg (mean ± SEM). Larvae in the infected group (no. 2) were fed with 10-μl Day 0 diet containing *M. plutonius* DAT606(CC3) at a final concentration of 1.4 × 10^7^ CFU/ml (Supplementary Table S1). Five larvae were randomly selected from each group daily and weighed. The larvae were also used for determination of bacterial loads in the larvae. Asterisks indicate a significant difference between control and DAT606(CC3)-infected groups (two-tailed Student *t*-test, *P* < 0.05). (**C**) Bacterial loads in infected larvae calculated by real-time PCR and presented as log_10_CFU/larva (mean ± SEM). Total no. of larvae used for each experiment is shown in Supplementary Table S1.

In experimental infection I, although the expected final concentration of DAT606(CC3) in the Day 0 diet (1.4–1.7 × 10^7^ CFU/ml) (Supplementary Table S1) was higher than that of the previous study (5 × 10^6^ CFU/ml)^4^, the survival rate of the DAT606(CC3)-infected group at day 5 pi (94.7%) was comparable to that of the control group (92.3%) (Fisher’s exact test, *P* = 1) (Fig. 1A). The average larval weight of the DAT606(CC3)-infected group was lighter than that of the control group at day 3 pi or later; however, no significant difference was observed between the two groups during days 2–5 pi (two-tailed Student *t*-test, *P* = 0.063–0.526) (Fig. 1B). The average bacterial loads at day 3 pi or later were 6.462 ± 0.069 log_10_CFU/larva or higher (Fig. 1C), which were also comparable to those of the previous study^4^. Although experimental conditions were slightly different from the previous study, the results were well reproduced, indicating that DAT606(CC3) does not show clear pathogenicity to honeybee larvae at least under these conditions, until day 5 pi.

However, at day 6 pi, the survival rate of the DAT606(CC3)-infected group (42.1%) decreased markedly and became significantly lower than that of the control group (89.7%) (Fisher’s exact test, *P* < 0.001) (Fig. 1A). In addition, the average larval weight of the DAT606(CC3)-infected group at day 6 pi (74.41 ± 11.53 mg) was significantly less than that of the control group (138.56 ± 17.97 mg) (two-tailed Student *t*-test, *P* = 0.017) (Fig. 1B). Therefore, one of the reasons for the previous unclear pathogenicity of DAT606(CC3) may be the short observation period.

### Experimental infection II-1: The influence of Na:K ratio in Day 0 diet on infected larvae

Artificial diets for honeybee larvae consist of D-glucose, D-fructose, yeast extract, royal jelly (RJ) and water (Table 2). Although the composition of RJ varies with seasonal and regional conditions, Na and K concentrations in RJ reported by Stocker *et al.* were 106–142 mg/kg and 2462–3120 mg/kg, respectively^13^. According to BD Bionutrients^TM^ Technical Manual^14^, Na and K concentrations in Bacto^TM^ Yeast Extract were 4900 mg/kg and 31950 mg/kg, respectively. Because the density of Diet A is about 1.1 mg/μl^11^,^12^, when these values are adopted, Na and K concentrations in Diet A are expected to be approximately 4.88–5.74 mM and 43.62–52.88 mM, respectively. In condition no. 2 and those reported previously^4^, *M. plutonius* suspension in saline (Na concentration is approximately 154 mM) were mixed with an equal volume of Diet A to prepare the Day 0 diet. Therefore, Na concentration in the Day 0 diet used for the experiments (approximately 79.44–79.87 mM) was considered to be higher than K concentration (approximately 21.81–26.44 mM). As reported^4^, DAT606(CC3) cannot grow on agar media under Na > K conditions; therefore, in experimental infection I and those reported previously^4^, DAT606(CC3) may not be able to grow well in the larval midguts at the early stage of infection, resulting in the delayed onset of EFB.

In order to investigate this possibility, we performed experimental infection II (Table 1). In condition nos 3 and 5, the Day 0 diet was prepared by mixing Diet A with the same volume of saline (no. 3) or *M. plutonius* suspension in saline (no. 5), so the expected Na concentration was higher than the K concentration. In condition nos 4 and 6, the Day 0 diet was prepared by mixing Diet A with the same volume of water (no. 4) or *M. plutonius* suspension in water (no. 6). Therefore, the expected Na concentration (approximately 2.44–2.87 mM) was considered to be lower than the K concentration (approximately 21.81–26.44 mM). For preparation of the Day 0 diets, *M. plutonius* in saline or water was mixed with an artificial diet within 10 min, and either saline or water did not affect the survival of *M. plutonius* in the suspension within the time frame (Supplementary Methods and Fig. S1). Larvae of the infected groups were fed with DAT606(CC3) by doses of 1.3–2.1 × 10^7^ CFU/ml, and all larvae were reared by rationed feeding according to the OECD guideline and guidance^11^,^12^ (Table 3 and Supplementary Table S1).

**Figure 2:**
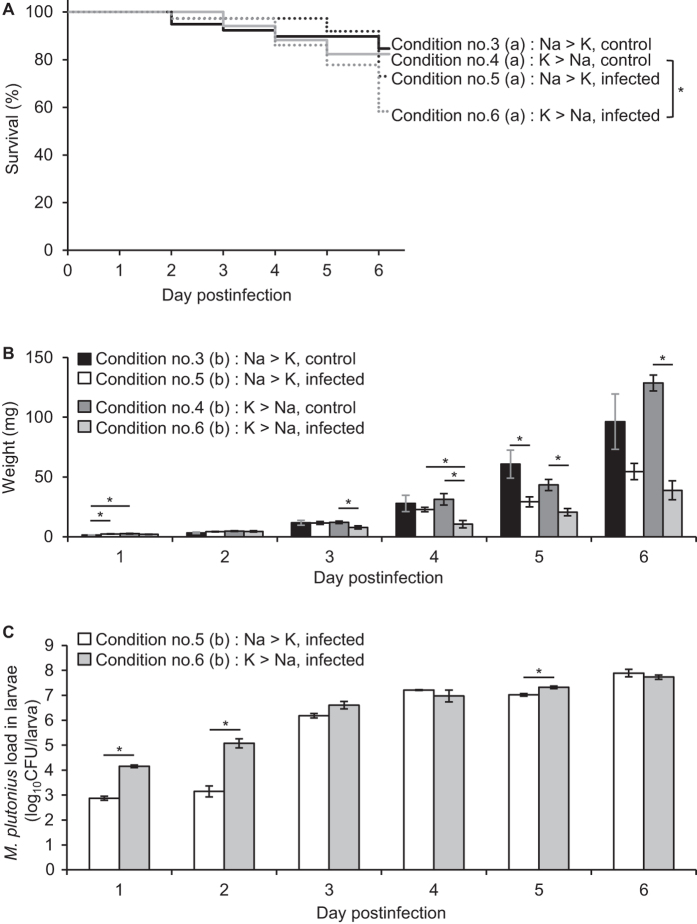
The influence of Na:K ratio in the Day 0 diet on symptoms of infected larvae (experimental condition nos 3–6). (**A**) Survival of control and infected larvae. Larvae in infected groups (nos 5 and 6) were fed with 10-μl Day 0 diet containing *M. plutonius* DAT606(CC3) at a final concentration of 1.7–2.1 × 10^7^ CFU/ml (Supplementary Table S1). Survival rates at day 6 pi were 84.6% (no. 3), 82.4% (no. 4), 73.0% (no. 5), and 58.3% (no. 6). Asterisk indicates a significant difference in the survival rate at day 6 pi (Fisher’s exact test, *P* < 0.05). (**B**) Larval weight presented as mg (mean ± SEM). Larvae in infected groups (nos 5 and 6) were fed with 10-μl Day 0 diet containing *M. plutonius* DAT606(CC3) at a final concentration of 1.3–1.4 × 10^7^ CFU/ml (Supplementary Table S1). Five larvae were randomly selected from each group daily and weighed. The larvae were also used for determination of bacterial loads in the larvae. Statistical differences were analysed between two control groups (nos 3 and 4), two infected groups (nos 5 and 6) and control and infected groups (between nos 3 and 5 and between nos 4 and 6). Asterisks indicate a significant difference (two-tailed Student *t*-test, *P* < 0.05). (**C**) Bacterial loads in infected larvae calculated by real-time PCR and presented as log_10_CFU/larva (mean ± SEM). Asterisks indicate a significant difference (two-tailed Student *t*-test, *P* < 0.05). Total no. of larvae used for each experiment is shown in Supplementary Table S1.

Under the non-infected control conditions (nos 3 and 4), Na:K ratio in the Day 0 diet did not affect both survival and growth of the larvae. Survival rates of condition nos 3 and 4 at day 6 pi were 84.6% and 82.4%, respectively, and no significant difference was observed in survival (Fisher’s exact test at day 6 pi, *P* = 1; log-rank test, *P* = 0.811) (Fig. 2A). In both groups, larval weight became heavier as the days went by, and except for day 1 pi, no significant difference was observed in the average larval weight between the two groups (two-tailed Student *t*-test, *P* = 0.069–0.871) (Fig. 2B).

When larvae were infected under the Na > K conditions (no. 5), the survival rate of the larvae at day 5 pi (91.9%) was again comparable to that in the control group (no. 3) (89.7%) (Fisher’s exact test, *P* = 1) (Fig. 2A). Although the survival rate of the infected group decreased to 73.0% at day 6 pi (Fig. 2A), no significant difference was observed between condition nos 3 and 5 (Fisher’s exact test at day 6 pi, *P* = 0.266; log-rank test, *P* = 0.276). The average larval weight of condition no. 5 at day 3 pi or later was lighter than that of the control larvae of condition no. 3; however, significant differences were not demonstrated between the two groups (two-tailed Student *t*-test, *P* = 0.122–0.937), except for day 5 pi (*P* = 0.035) (Fig. 2B). These results further suggest that it is difficult for DAT606(CC3) to produce clear disease in honeybee larvae when larvae are infected with Na-rich food.

In contrast, when larvae were infected under K > Na conditions (no. 6), the influence of DAT606(CC3) infection was more clearly observed. The survival rate of the larvae at day 6 pi under experimental condition no. 6 (58.3%) was significantly lower than that of the corresponding control group (no. 4, 82.4%) (Fisher’s exact test, *P* = 0.038) and also tended to be lower than that of the larvae infected under Na-rich conditions (no. 5, 73.0%) (Fig. 2A). DAT606(CC3) infection under K-rich conditions also apparently influenced larval growth. The increase of larval weight under condition no. 6 was significantly suppressed at day 3 pi or later compared with the control group (no. 4) (two-tailed Student *t*-test, *P* ≤ 0.033) (Fig. 2B). At day 3 pi or later, the average larval weight of condition no. 6 was also lighter than that of condition no. 5, and a significant difference was observed at day 4 pi (two-tailed Student *t*-test, *P* = 0.01) (Fig. 2B). Moreover, in the multiple regression model adjusted by days pi, Na:K ratio showed a significant effect on the larval weight gain, that is, DAT606(CC3) infection with Na-rich food (no. 5) allowed larvae to gain more weight than infection with K-rich food (no. 6) (*P* = 0.003, coefficient = 6.73).

In both infected groups, ingested DAT606(CC3) was proliferated in the larvae (Fig. 2C). However, at the early stage of infection (days 1–2 pi), the average bacterial loads under condition no. 5 were significantly lower than those of condition no. 6 (two-tailed Student *t*-test, *P* < 0.001 at day 1 pi and *P* = 0.01 at day 2 pi) (Fig. 2C). By the multiple regression model adjusted by days pi, Na-rich food (no. 5) was also shown to inhibit bacterial proliferation in larvae compared with K-rich food (no. 6) (*P* < 0.001, coefficient = −1.36). It is noteworthy that, in liquid culture media, the proliferation of DAT606(CC3) was also inhibited under Na-rich conditions. As shown in Supplementary Fig. S2, the concentration of viable DAT606(CC3) increased 27.57 ± 1.33 times in the K-rich medium [KSBHI broth (BHI broth with 1% soluble starch and 150 mM KH_2_PO_4_), Na/K  =  0.68] after 24-h incubation at 34 °C ± 0.5 °C under anaerobic conditions; however, in the Na-rich medium [kSBHI broth (BHI broth with 1% soluble starch and 13.5 mM KH_2_PO_4_), Na/K  =  3.31], the DAT606(CC3) concentration increased only 2.65 ± 0.22 times after 24 h, and the growth of the strain between the two media was significantly different [two-tailed Welch’s *t*-test (Supplementary Methods), *P* < 0.001].

These results strongly support the above hypothesis and suggest another reason for the previously reported unclear clinical symptoms in larvae infected with DAT606(CC3)^4^. That is, in the previous study, because larvae were fed with Na-rich food for the first 24 h^4^, larval gut contents were in high-Na conditions at an early stage of infection, and this delayed proliferation of the strain in the midguts, resulting in delayed onset of EFB and unclear clinical symptoms in the experiments. Therefore, for better evaluation of *M. plutonius* virulence, larvae should be infected using bacteria suspended in a high-K and low-Na diet.

### Experimental infection II-2: The influence of dilution of the Day 0 diet on larvae

Under the experimental conditions tested so far, the Day 0 diet was prepared by diluting Diet A with an equal volume of saline, water or bacterial suspension (Table 1), so larvae were fed with jejune diets for the first 24 h compared with the ideal rearing conditions^11^,^12^, and this may influence the survival and growth of larvae. To investigate this possibility, we tested condition nos 7 and 8. In these conditions, the Day 0 diet was prepared by mixing nine volumes of Diet A′ with one volume of saline (no. 7) or *M. plutonius* suspension in saline (no. 8) (Tables 1 and 2); therefore, larvae at day 0 can be fed with a more nutritious diet (i.e., undiluted diet) than those of condition nos 1–6. Because the expected K concentration (approximately 43.62–52.88 mM) in the undiluted diets was still higher than the Na concentration (approximately 20.28–21.14 mM), we compared the results with those of condition nos 4 and 6.

**Figure 3:**
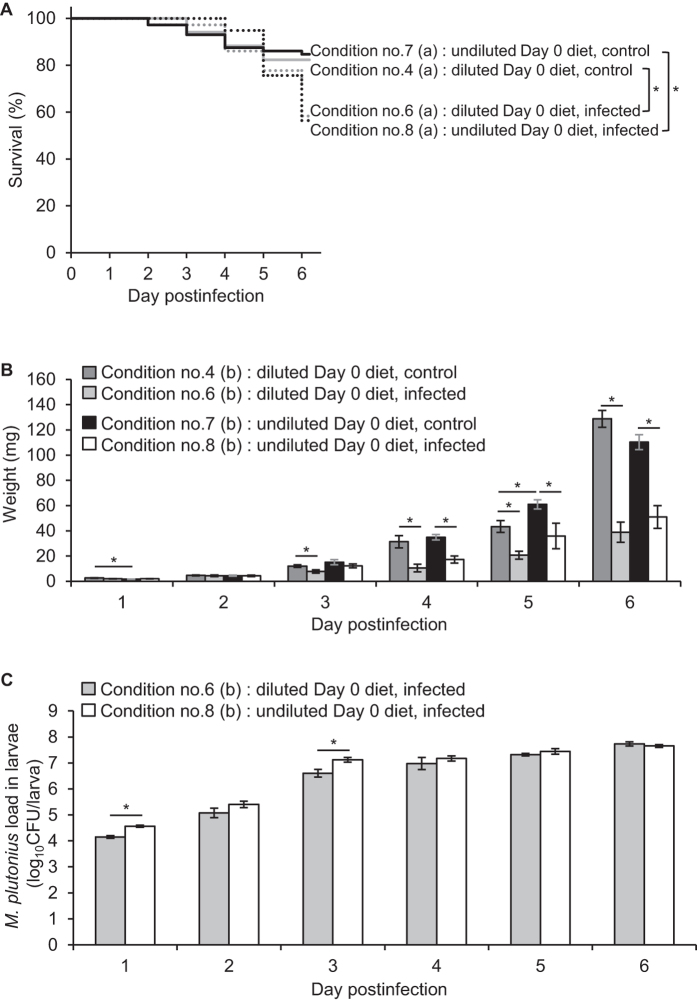
The influence of dilution of the Day 0 diet on the survival and growth of larvae (experimental condition nos 4 and 6–8). (**A**) Survival of control and infected larvae. Larvae in infected groups (nos 6 and 8) were fed with 10-μl Day 0 diet containing *M. plutonius* DAT606(CC3) at a final concentration of 1.3–2.1 × 10^7^ CFU/ml (Supplementary Table S1). Survival rates at day 6 pi were 82.4% (no. 4), 58.3% (no. 6), 84.7% (no. 7) and 56.4% (no. 8). Asterisks indicate a significant difference in the survival rate at day 6 pi (Fisher’s exact test, *P* < 0.05). (**B**) Larval weight presented as mg (mean ± SEM). Larvae in infected groups (nos 6 and 8) were fed with 10-μl Day 0 diet containing *M. plutonius* DAT606(CC3) at a final concentration of 1.3–1.8 × 10^7^ CFU/ml (Supplementary Table S1). Five larvae were randomly selected from each group daily and weighed. The larvae were also used for determination of bacterial loads in larvae. Statistical differences were analysed between two control groups (nos 4 and 7), two infected groups (nos 6 and 8) and control and infected groups (between nos 4 and 6 and between nos 7 and 8). Asterisks indicate a significant difference (two-tailed Student *t*-test, *P* < 0.05). (**C**) Bacterial loads in infected larvae calculated by real-time PCR and presented as log_10_CFU/larva (mean ± SEM). Asterisks indicate a significant difference (two-tailed Student *t*-test, *P* < 0.05). Total no. of larvae used for each experiment is shown in Supplementary Table S1.

As shown in Fig. 3, dilution of the Day 0 diet did not influence the survival or growth of the control larvae. Survival rates of the control groups at day 6 pi were 82.4% (no. 4) and 84.7% (no. 7), and no significant difference was observed (log-rank test, *P* = 0.790; Fisher’s exact test at day 6 pi, *P* = 0.781) (Fig. 3A). Although some differences were observed in the average larval weight at days 1 and 5 pi (two-tailed Student *t*-test, *P* = 0.010–0.018), the dilution did not show any significant effect on weight gain during the experiments when analysed with the multiple regression model adjusted by days pi (*P* = 0.76).

No significant difference in survival was shown between the two infected groups by both Fisher’s exact test at day 6 pi (the survival rates of nos 6 and 8 were 58.3% and 56.4%, respectively, *P* = 1) and log-rank test (*P* = 0.949). Although the average larval weight was not significantly different between the two groups by two-tailed Student *t*-test (*P* = 0.063–0.993), the multiple regression model adjusted by days pi indicated that larvae fed with the undiluted diet (no. 8) gained more weight than those fed with the diluted diet (no. 6) (*P* = 0.02, coefficient = 6.40). As expected from the Na:K ratio of the undiluted Day 0 diet, DAT606(CC3) proliferated in the larvae soon after the ingestion under condition no. 8, and the bacterial loads were comparable to or higher (two-tailed Student *t*-test, *P* < 0.001 at day 1 pi and *P* = 0.038 at day 3 pi; the multiple regression model adjusted by days pi, *P* < 0.001, coefficient = 0.57) than those of condition no. 6. These results suggested that dilution of the Day 0 diet does not have a substantial impact on the results of experimental infections. However, feeding larvae with a nutritious diet for the first 24 h appears to somewhat promote the growth of both infected larvae and bacteria in the larvae. One of the pathogenic effects associated with EFB is the competition for nutrients between the infected larva and the pathogen, resulting in the starvation of larvae^15^. Under condition no. 8, the starvation of the infected larvae might be somewhat moderated, resulting in a slight alleviation of the growth inhibition.

Even when undiluted diet was used, the disease was clearly caused by DAT606(CC3). The survival of the infected larvae (no. 8) was significantly lower than that of the control group (no. 7) (Fisher’s exact test at day 6 pi, *P* < 0.001; log-rank test, *P* < 0.001) (Fig. 3A). The growth of the infected larvae was also significantly impeded compared with that of the control larvae (two-tailed Student *t*-test during days 4–6 pi, *P* ≤ 0.048; the multiple regression model adjusted by days pi, *P* < 0.001, coefficient = −17.46) (Fig. 3B). Because feeding with undiluted Day 0 diet is closer to the rearing conditions described in the OECD guidelines^11^,^12^ than feeding larvae with a diluted diet, we used undiluted Day 0 diet for the next experiments.

### Experimental infection III: Virulence of *M. plutonius* strains with different genetic backgrounds

In the previous study by our group, atypical (CC12) *M. plutonius* strains killed most of the experimentally infected larvae within five days, while typical (CC3 and CC13) strains hardly affected larvae under the conditions tested^4^. However, as demonstrated above, because the previous experimental conditions were not appropriate for the growth of typical strains in larvae, differences in their virulence could not be evaluated correctly. Therefore, we selected strains DAT606(CC3), DAT561 of CC12 [DAT561(CC12)] and DAT585 of CC13 [DAT585(CC13)] as representatives of each CC, and investigated their virulence again under the improved experimental conditions.

**Figure 4:**
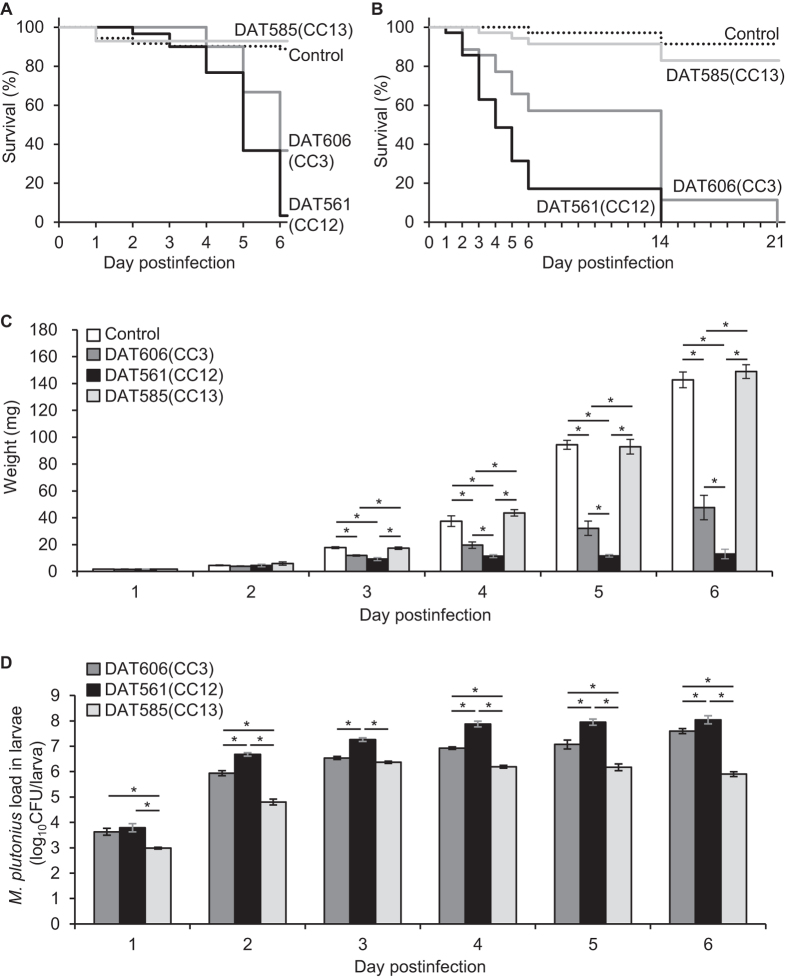
Evaluation of virulence of *M. plutonius* strains belonging to different CCs under the improved experimental conditions. (**A**) Survival of control and infected larvae (condition nos 9–12). (**B**) Survival of control and infected larvae (condition nos 13–16). Larvae in infected groups (nos 10–12 and 14–16) were fed with 10-μl Day 0 diet containing *M. plutonius* at a final concentration of 2.5 × 10^5^ to 3.6 × 10^6^ CFU/ml (Supplementary Table S1). (**C**) Larval weight presented as mg (mean ± SEM) (condition nos 13–16). Larvae in infected groups (nos 14–16) were fed with 10-μl Day 0 diet containing *M. plutonius* at a final concentration of 2.5–5.7 × 10^5^ CFU/ml (Supplementary Table S1). Four to six larvae were randomly selected from each group daily and weighed. The larvae were also used for determination of bacterial loads in the larvae. Asterisks indicate a significant difference (two-tailed Student *t*-test, *P* < 0.05). (**D**) Bacterial loads in larvae calculated by real-time PCR and presented as log_10_CFU/larva (mean ± SEM) (condition nos 14–16). Asterisks indicate a significant difference (two-tailed Student *t*-test, *P* < 0.05). Total no. of larvae used for each experiment is shown in Supplementary Table S1.

In the first set of experiments (nos 9–12), larvae in infected groups were fed with lower doses of *M. plutonius* than in experimental infections I and II (6.0 × 10^5^ [DAT606(CC3)], 6.7 × 10^5^ [DAT561(CC12)] and 3.6 × 10^6^ [DAT585(CC13)] CFU/ml) (Supplementary Table S1). Nevertheless, DAT606(CC3) caused disease in the larvae, and the survival rate at day 6 pi was 36.7% (Fig. 4A), demonstrating that this dose is sufficiently high for DAT606(CC3) to cause EFB in honeybee larvae. In the previous study^4^, DAT561(CC12) exhibited high pathogenicity and killed more than 90% of tested larvae within five days. In this study, DAT561(CC12) also exhibited high pathogenicity and killed larvae more rapidly than DAT606(CC3) (Fisher’s exact test at day 6 pi, *P* = 0.003; log-rank test, *P* = 0.001) (Fig. 4A). One DAT561(CC12)-infected larva still survived at day 6 pi; however, it was quite small and died the next day. In contrast, DAT585(CC13) did not cause EFB even under the improved conditions. The mortality of the DAT585(CC13)-infected group was significantly lower than that of the DAT606(CC3)- and DAT561(CC12)-infected groups (Fisher’s exact test at day 6 pi, *P* < 0.001; log-rank test, *P* < 0.001) and was at the same level as the control group (Fisher’s exact test at day 6 pi, *P* = 0.549; log-rank test, *P* = 0.458) (Fig. 4A).

To confirm these results, we fed larvae again with the three strains at doses of 2.5–5.7 × 10^5^ CFU/ml (Supplementary Table S1; nos 13–16) and observed survival until day 21 pi. In the second trials, both DAT606(CC3) and DAT561(CC12) killed all the tested bees by the end of the experiments, and again, death was more rapid from DAT561(CC12) than DAT606(CC3) (log-rank test, *P* < 0.001) (Fig. 4B). No DAT561(CC12)-infected larvae pupated. In contrast, part of the DAT606(CC3)-infected larvae pupated. Larval growth was also inhibited by the two strains, but weight gain was more strongly inhibited by DAT561(CC12) than DAT606(CC3) (two-tailed Student *t*-test during days 4–6 pi, *P* = 0.005–0.014) (Fig. 4C). In contrast, more than 80% of the DAT585(CC13)-infected bees survived and had emerged by day 21 pi. The low mortality was comparable to that of the control group (Fisher’s exact test at day 21 pi, *P* = 0.477; log-rank test, *P* = 1) (Fig. 4B). In addition, the average weight of DAT585(CC13)-infected larvae reached 148.95 ± 5.14 mg at day 6 pi, which was comparable to that of the control larvae (142.79 ± 5.89 mg at day 6 pi; two-tailed Student *t*-test, *P* = 0.449) (Fig. 4C). The multiple regression model adjusted by days pi also supported no significant effect of DAT585(CC13) infection on larval weight gain (*P* = 0.785). In contrast, DAT606(CC3) and DAT561(CC12) were shown to inhibit weight increase remarkably compared to the control larvae (*P* < 0.001, coefficient = −32.68 and −42.67, respectively) (Fig. 4C). These results demonstrated that, under the tested conditions, DAT561(CC12) was the most virulent followed by DAT606(CC3) and that DAT585(CC13) was avirulent to honeybee broods.

In larvae, DAT561(CC12) proliferated the most vigorously among the three strains. The average bacterial loads were always significantly higher than those of the other two strains at day 2 pi or later (two-tailed Student *t*-test, *P* ≤ 0.034) and reached 8.041 ± 0.158 log_10_CFU/larva at day 6 pi (Fig. 4D). Although the proliferation potency of DAT606(CC3) seemed to be inferior to DAT561(CC12), the average bacterial loads of DAT606(CC3) also increased throughout the larval stage and reached 7.593 ± 0.098 log_10_CFU/larva at day 6 pi (Fig. 4D). On the other hand, the average bacterial loads of DAT585(CC13) reached a peak at day 3 pi (6.369 ± 0.045 log_10_CFU/larva), and except for day 3 pi, the average bacterial loads were always significantly lower than those of DAT606(CC3) (two-tailed Student *t*-test, *P* ≤ 0.003) (Fig. 4D). The proliferation potency was confirmed in the multiple regression model adjusted by days pi. Compared with DAT585(CC13), both DAT561(CC12) and DAT606(CC3) had significantly well proliferated in larvae (*P* < 0.001), and the coefficients were 3.56 and 2.09, respectively. These results were consistent with the results of the larval growth and survival, suggesting that bacterial proliferation in larvae is an important factor in the pathogenesis of EFB.

In most of the EFB studies reported to date, *M. plutonius* strains used for experimental infections were not characterised^6^,^7^,^8^,^9^, and thus it was unknown whether differences in the genotypes of the strains influenced the results of the experimental infections. In this study, we suggested for the first time that virulence at the brood level is different among *M. plutonius* strains with different genetic backgrounds. Budge *et al.* reported that honeybee colonies infected with CC3 strains contained more diseased brood frames than those infected with CC13 strains and that a significantly higher proportion of larvae were diseased on brood frames infected with CC3 strains compared with CC13 strains^2^. Although no significant differences in the proportion of destroyed colonies and diseased brood were observed between CC3 and CC12 or between CC12 and CC13^2^, that data including the value of diseased ranking calculated from the proportion of the diseased brood imply that CC3 is the most virulent group in the field followed by CC12 and then CC13. Interestingly, this order is inconsistent with the order of the individual-insect level virulence of the strains tested in this study, that is, DAT561(CC12) was the most virulent followed by DAT606(CC3) and then DAT585(CC13). Since we tested only a single strain each from the CCs, further study is needed to verify this tendency. However, because other CC12 strains DAT351 and DAT573 also killed most of the tested larvae within five days in the previous study^4^, extremely high virulence against honeybee larvae is likely to be a common characteristic of CC12 strains.

As suggested from the results of the DAT585 infection, the virulence of CC13 strains for the individual bee may be remarkably lower than those of the other CCs. However, because many CC13 strains including DAT585(CC13) were isolated from diseased larvae^2^,^4^, CC13 strains are also considered to have the ability to cause EFB in the field. Therefore, we cannot rule out the possibility that the avirulent phenotype of DAT585(CC13) resulted from a decline in virulence due to the *in vitro* subcultures. As outbreaks of EFB appear to be linked to stress conditions, such as a lack of food or water^15^, DAT585(CC13) may be able to cause EFB even under *in vitro* conditions by restricting the diet. Alternatively, DAT585(CC13) may require secondary invaders to produce symptoms in larvae. Indeed, in a previous study by Bailey^6^, EFB could not be produced in honeybee colonies by *M. plutonius* alone but could be successfully produced by mixed cultures of *M. plutonius* and *Achromobacter eurydice* (a common secondary invader of EFB). Giersch *et al.* also reported that the combination of *M. plutonius* and *P. alvei* (another common secondary invader) was required to reliably produce typical symptoms seen in field EFB cases^8^, although the genotypes of *M. plutonius* strains used in these studies were unknown.

As these possibilities suggest, further analysis is necessary to verify whether the virulence of the strains observed under the *in vitro* conditions accurately reflected virulence in the field; however, the improved experimental infection method of *M. plutonius* and the three strains, which show different degrees of virulence against honeybee larvae, will be very useful tools for future EFB studies. In particular, the combination of these tools with recently developed gene manipulation techniques for *M. plutonius*^5^,^16^ will lead to further elucidation of the pathogenic mechanisms of EFB.

## Methods

### *M. plutonius* strains and preparation of inocula

*M. plutonius* strains DAT561(CC12), DAT585(CC13) and DAT606(CC3) were used in this study. All the strains were isolated from diseased larvae of *A. mellifera* with clinical signs of EFB in Japan^4^. Strains DAT585(CC13) and DAT606(CC3) were called typical *M. plutonius* in the previous study^4^ and were assigned into ST26 of CC13 and ST3 of CC3, respectively, by MLST^3^. Strain DAT561(CC12) was called atypical *M. plutonius* in the previous study^4^ and was assigned into ST12 of CC12^3^. DAT606(CC3) was used as a representative strain in the experiments I and II (Table 1) in order to improve experimental conditions, and all the three strains were used in experiment III to compare their virulence under the improved experimental conditions. All the strains were cultured on KSBHI agar plates^4^ at 37 °C for five days under anaerobic conditions, suspended in sterile saline or water, and adjusted to appropriate concentrations by diluting the suspensions with sterile saline or water. Then, the suspension was mixed with an artificial diet according to the formulas described in Table 1. The mixture (Day 0 diet) was used as inocula. Final bacterial concentration in each inocula was determined by plating serial dilutions of the Day 0 diet onto KSBHI agar plates and counting colonies on the plates after incubation of the plates at 37 °C for five days under anaerobic conditions. The results are shown in Supplementary Table S1.

### Experimental infection

European honeybees (*A. mellifera*) maintained in the Research Institute for Animal Science in Biochemistry and Toxicology, Sagamihara, Japan, were used in this study. All the used colonies were clinically healthy, and the fertility of the queens was normal. To obtain young larvae, the queens were confined for approximately 24 h in their own colony in an exclusion case containing an empty comb. Seventy-two hours after the encaging was started, less than 24-h-old larvae hatched in the comb were grafted onto RJ in sterile Petri dishes by a grafting tool. The larvae were then randomly divided into test groups, and each larva was fed with 10 μl of Day 0 diet with or without *M. plutonius* according to the experimental conditions described in Table 1 and Supplementary Table S1. Each larva was reared in a grafting cell placed into a well of a 48-well cell culture plate. The culture plates were kept in a desiccator with a relative humidity of 96% and incubated at 34 °C ± 0.5 °C until day 6 pi. Larvae were fed once a day until day 5 pi. Daily rations and formulas of the artificial diet are shown in Tables 2 and 3. In experiment III (condition nos 13–16), the culture plates were further incubated in a desiccator at 34 °C ± 0.5 °C with a relative humidity of 80%. On day 14 pi, each plate was transferred into an emergence box in a desiccator and incubated at 34 °C ± 0.5 °C with a relative humidity of 80% until day 21 pi. To avoid mechanical damage by grafting, larvae were reared in the same wells during the test period.

In most of the experimental conditions, two groups of larvae [groups (a) and (b)] were tested. Group (a) was used to determine mortality. Group (b) was used to measure larval weight and bacterial loads in the larvae. Four to six larvae per day were randomly selected from group (b), weighted and stored in a deep freezer at −80 °C until use for determination of bacterial loads by real-time PCR. The total no. of larvae used for each experiment is shown in Supplementary Table S1.

### Real-time PCR

Bacterial loads in *M. plutonius*-infected larvae were evaluated using quantitative real-time PCR. On the basis of the results of comparative genomic analyses reported previously^17^, we selected Na^+^/H^+^ antiporter gene [MPTP_RS01890 in ATCC 35311 (old locus tag: MPTP_0420), GenBank accession no. NC_015516.1] and Fur family transcriptional regulator gene [MPD5_RS04175 in DAT561(CC12) (old locus tag: MPD5_0863), GenBank accession no. NC_016938.1] as targets to detect typical [DAT585(CC13) and DAT606(CC3)] and atypical [DAT561(CC12)] *M. plutonius* strains, respectively, and designed two primer sets for the real-time PCR by using Primer3 (http://bioinfo.ut.ee/primer3-0.4.0) (Table 4). The specificity of the primers against DNA sequences of related bacteria available in the GenBank database was assessed by BLAST search (http://blast.ncbi.nlm.nih.gov/Blast.cgi).

Genomic DNAs of bacteria in infected larvae as well as those cultured artificially on KSBHI agar were extracted by the DNeasy Blood & Tissue kit (Qiagen) according to the manufacturer’s instructions designed for Gram-positive bacteria. For extraction of DNA from bee larvae with a weight not more than 20 mg, a larva was homogenised with 200 μl of the enzymatic lysis buffer (ELB) [20 mM Tris-HCl (pH 8.0), 2 mM sodium EDTA, 1.2% Triton X-100 and 20 mg/ml lysozyme], and all the homogenate was used for DNA extraction by the kit. For larvae with a weight more than 20 mg and 50 mg, 500 μl and 1,000 μl of ELB, respectively, were added to a single larva, and 100 μl of the larval homogenate was used for DNA extraction. DNA was eluted with 200 μl of the elution buffer in the kit, and 1 μl of the extracted DNA was used for each reaction. The real-time PCR was carried out by the QuantiTect SYBR^®^ Green PCR kit (Qiagen) according to the manufacturer’s instructions, except that the final concentration of primers was 2 μM. Amplifications were run in a Thermal Cycler Dice^®^ Real Time System (TaKaRa Bio Inc., Kusatsu, Japan) using the following program: 15 min at 95 °C and 45 cycles of 15 sec at 95 °C, 30 sec at 53 °C, and 30 sec at 72 °C. Each sample was measured in duplicate. Under the above conditions, controls without template DNA were negative in all PCR runs, and neither primer set amplified any specific PCR products from non-infected larvae and *Enterococcus faecalis*, a common secondary invader associated with EFB and taxonomically related to *M. plutonius* (Supplementary Fig. S3). Because the presence of the larval tissue and DNA did not affect extraction of *M. plutonius* DNA and amplification of the target genes (Supplementary Fig. S4), genomic DNA extracted from a known concentration of *M. plutonius* suspension in saline was included in each real-time PCR run to prepare the standard curve for *M. plutonius* quantification.

### Statistical analysis

The differences in the survival of tested larvae throughout the experiments were analysed by the log-rank test with multiple comparisons (Bonferrini correction) and those at the specified day pi were evaluated by the Fisher’s exact test. The larval weight and bacterial loads in infected larvae were expressed as mean ± standard errors and analysed by two-tailed Student *t*-test to assess the effect of Na:K ration, dilution of Day 0 diet, infection status or *M. plutonius* strains, respectively. Considering the days pi, multiple regression analyses were also performed for these data. For all tests, a value of *P* < 0.05 was considered as the threshold for significance.

## Additional Information

**How to cite this article**: Nakamura, K. *et al.* Virulence Differences among *Melissococcus plutonius* Strains with Different Genetic Backgrounds in *Apis mellifera* Larvae under an Improved Experimental Condition. *Sci. Rep.* **6**, 33329; doi: 10.1038/srep33329 (2016).

## Supplementary Material

Supplementary Information

## Figures and Tables

**Table 1:** Conditions of experimental infection.

Experiments	Condition	Formulas of Day 0 diet			*M. plutonius* strain	Expected Na:K ratio in Day 0 diet	Survival rate at day 5 pi	Survival rate at day 6 pi
		Diet A/A′	Saline or bacterial suspension in saline	Water or bacterial suspension in water				
I	No. 1	5 ml*	5 ml	0 ml	non-infected control	Na > K	92.3%	89.7%
	No. 2	5 ml*	5 ml†	0 ml	DAT606(CC3)	Na > K	94.7%	42.1%
II	No. 3	5 ml*	5 ml	0 ml	non-infected control	Na > K	89.7%	84.6%
	No. 4	5 ml*	0 ml	5 ml	non-infected control	K > Na	82.4%	82.4%
	No. 5	5 ml*	5 ml†	0 ml	DAT606(CC3)	Na > K	91.9%	73.0%
	No. 6	5 ml*	0 ml	5 ml¶	DAT606(CC3)	K > Na	77.8%	58.3%
	No. 7	9 ml**	1 ml	0 ml	non-infected control	K > Na	86.1%	84.7%
	No. 8	9 ml**	1 ml†	0 ml	DAT606(CC3)	K > Na	75.6%	56.4%
III	No. 9	9 ml**	1 ml	0 ml	non-infected control	K > Na	90.3%	88.9%
	No. 10	9 ml**	1 ml†	0 ml	DAT606(CC3)	K > Na	66.7%	36.7%
	No. 11	9 ml**	1 ml†	0 ml	DAT561(CC12)	K > Na	36.7%	3.3%
	No. 12	9 ml**	1 ml†	0 ml	DAT585(CC13)	K > Na	92.9%	92.9%
	No. 13	9 ml**	1 ml	0 ml	non-infected control	K > Na	100.0%	97.1%
	No. 14	9 ml**	1 ml†	0 ml	DAT606(CC3)	K > Na	65.7%	57.1%
	No. 15	9 ml**	1 ml†	0 ml	DAT561(CC12)	K > Na	31.4%	17.1%
	No. 16	9 ml**	1 ml†	0 ml	DAT585(CC13)	K > Na	94.3%	91.4%

Formulas of diets A and A′ are listed in Table 2.

^*^Diet A was used for preparation of the Day 0 diet.

^**^Diet A′ was used for preparation of the Day 0 diet. Because the ratio of water was reduced in diet A′ compared with diet A (Table 2), final concentrations of D-glucose, D-fructose, yeast extract, and royal jelly in the Day 0 diet for condition nos 7–16 were almost the same as those in diet A.

^†^*M. plutonius* suspension in saline.

^¶^*M. plutonius* suspension in water.

**Table 2:** Formulas of the artificial diet for honeybee larvae used in the experimental infection.

Contents	Artificial diet			
	A	A′	B	C
D-glucose	6 g	6 g	7.5 g	9 g
D-fructose	6 g	6 g	7.5 g	9 g
Yeast extract	1 g	1 g	1.5 g	2 g
Royal jelly	50 g	50 g	50 g	50 g
Sterile H_2_O	37 g	27 g	33.5 g	30 g
Total	100 g	90 g	100 g	100 g

**Table 3:** Daily rations of the artificial diet.

Day postinfection	Artificial diet	Amount (μl)/larva	
		*ad libitum* feeding (condition nos 1 and 2)	Rationed feeding (condition nos 3–16)
0	Day 0 diet	10	10
1	Diet A	Sufficient quantity	10
2	Diet B	Sufficient quantity	20
3	Diet C	Sufficient quantity	30
4	Diet C	Sufficient quantity	40
5	Diet C	Sufficient quantity	50

Larvae were not fed on day 6 postinfection. Formulas of artificial diet are listed in Table 2, and the Day 0 diet was made according to the recipe described in Table 1. Under the *ad libitum* feeding conditions, a sufficient quantity of the diet was added to each well every day to prevent any shortage, so the total amount of diet given to each larva differed among larvae.

**Table 4:** *M. plutonius*-specific real-time PCR primers designed in this study.

Target strain	Locus tag of target genes	Primer	Oligonucleotide sequence (5′-3′)	PCR product size
DAT585(CC13) and DAT606(CC3)	MPTP_0420 (MPTP_RS01890)*	Mp-Trt-F	GTTCTTAGGTGGTAGCTTAGGC	97 bp
		Mp-Trt-R	GGCCATTTCCCCTCTAGAGATC	
DAT561(CC12)	MPD5_0863 (MPD5_RS04175)	Mp-Art-F	CCATCCAACGGCAGATGAAATC	146 bp
		Mp-Art-R	CAAACCGACTGGATGTATCTCC	

^*^Locus tag in *M. plutonius* ATCC 35311.
