# Supplementary material for: Virulence Differences among Melissococcus plutonius Strains with Different Genetic Backgrounds in Apis mellifera Larvae under an Improved Experimental Condition
Source: Sci Rep. 2016 Sep 14;6:33329. doi: 10.1038/srep33329 (PMC5022037; doi:10.1038/srep33329)
Supplement: Supplementary Information [file srep33329-s1.pdf]

Supplementary Information

Virulence Differences among *Melissococcus plutonius* Strains with Different Genetic Backgrounds in *Apis mellifera* Larvae under an Improved Experimental Condition

Keiko Nakamura[1], Yuko Yamazaki[1], Akiyo Shiraishi[1], Sota Kobayashi[2], Mariko Harada[1], Mikio Yoshiyama[3], Makoto Osaki[4], Masatoshi Okura[4], Daisuke Takamatsu[4,5]

[1] Research and Business Promotion Division, Research Institute for Animal Science in Biochemistry and Toxicology, Sagamihara, Kanagawa 252-0132, Japan

[2] Division of Viral Diseases and Epidemiology, National Institute of Animal Health, National Agriculture and Food Research Organization, Tsukuba, Ibaraki 305-0856, Japan

[3] Division of Animal Breeding and Reproduction Research, Institute of Livestock and Grassland Science, National Agriculture and Food Research Organization, Tsukuba, Ibaraki 305-0901, Japan

[4] Division of Bacterial and Parasitic Diseases, National Institute of Animal Health, National Agriculture and Food Research Organization, Tsukuba, Ibaraki 305-0856, Japan

[5] The United Graduate School of Veterinary Sciences, Gifu University, Gifu, Gifu 501-1193, Japan

Supplementary Methods

Survival of *M. plutonius* in water and saline. In this study, for preparation of inocula for experimental infections, *M. plutonius* was suspended in sterile water or saline at room temperature and then mixed with an artificial diet within 10 min. In order to know the influence of the suspension media (in particular, hypoosmotic shock in water) on the *M. plutonius* survival, we investigated the survival of DAT606(CC3) in water and saline.

DAT606(CC3) cultured on KSBHI agar [Brain Heart Infusion (BHI) agar with 1% soluble starch and 150 mM KH_2PO_4] plates at 37ºC for five days under anaerobic conditions was suspended in KSBHI broth (BHI broth with 1% soluble starch and 150 mM KH_2PO_4) (optical density at 600 nm of 1.0). Bacterial cells in 500 µl of the suspension were collected by centrifugation (15,000 rpm, 5 min), and the supernatant was removed completely. Collected bacterial cells were then suspended with 500 µl of sterile water or saline in microtubes. Immediately after the suspension and after incubation for 10, 30 and 60 min at room temperature (25ºC), 10 µl of the suspension was collected from each tube, and serial dilutions of the suspensions were made with KSBHI broth. Bacterial survival in the suspension was investigated by plating the serial dilutions onto KSBHI agar plates and counting colonies on the plates after incubation of the plates at 35-37ºC for four days under anaerobic conditions. Data were collected from five independent suspensions. The differences in the concentration of viable *M. plutonius* were analysed by repeated ANOVA.

Growth of *M. plutonius* in liquid culture media with known concentrations of Na and K.

In order to investigate the influence of the Na:K ratio in liquid culture media on the growth of *M. plutonius*, we cultured DAT606(CC3) in kSBHI broth (BHI broth with 1% soluble starch and 13.5 mM KH_2PO_4) and KSBHI broth (BHI broth with 1% soluble starch and 150 mM KH_2PO_4). Na and K concentrations in BHI broth determined by flame atomic absorption

spectrometry using Shimadzu Atomic Absorption Spectrophotometer AA-6800 (the combustion gases: air and acetylene, the wavelengths: Na 589.0 nm and K 766.5 nm) in Prophoenix Division, Food and Life-Science Laboratory, Idea Consultants, Inc. (Osaka, Japan) were 2,700 mg/L and 860 mg/L, respectively. Therefore, Na and K concentrations in kSBHI broth were considered to be 117.44 mM and 35.50 mM, respectively, and the Na:K ratio (Na/K = 3.31, Na > K condition) was comparable to those of the Day 0 diet for experimental condition nos. 1, 2, 3 and 5. On the other hand, Na and K concentrations in KSBHI broth were considered to be 117.44 mM and 172.00 mM, respectively (Na/K = 0.68, K > Na condition).

DAT606(CC3) cultured on KSBHI agar plates at 37ºC for five days under anaerobic conditions was suspended in KSBHI broth (optical density at 600 nm of 0.8). Four ml of kSBHI or KSBHI broth was inoculated with 40 µl of the suspension. Immediately after the inoculation (0 h) and after incubation for 24 h at 34ºC ± 0.5ºC under anaerobic conditions, 200 µl of the culture was collected from each tube, and serial dilutions of the samples were made with KSBHI broth. Bacterial growth in the culture media was investigated by plating the serial dilutions onto KSBHI agar plates and counting colonies on the plates after incubation of the plates at 37ºC for four days under anaerobic conditions. Data were collected from five independent cultures, and the results were expressed as fold-increase relative to the concentration of viable bacteria at 0 h (mean ± SEM). The difference in the increase of DAT606(CC3) concentration between the two culture media was compared by two-tailed Welch's *t*-test.

Supplementary Table S1. Additional information of experimental conditions.

Experimental Infection	Condition (group)*	Measurement items	No. of larvae used	*M. plutonius* strain	Final concentration of *M. plutonius* in Day 0 diet (CFU/ml)	Expected Na:K ratio in Day 0 diet	Feeding regime**
I	No. 1 (a)	Survival	39	non-infected control	0	Na > K	*ad libitum*
	No. 1 (b)	Larval weight, bacterial load	30	non-infected control	0	Na > K	*ad libitum*
	No. 2 (a)	Survival	38	DAT606(CC3)	1.7×10^7	Na > K	*ad libitum*
	No. 2 (b)	Larval weight, bacterial load	30	DAT606(CC3)	1.4×10^7	Na > K	*ad libitum*
II	No. 3 (a)	Survival	39	non-infected control	0	Na > K	Rationed
	No. 3 (b)	Larval weight, bacterial load	30	non-infected control	0	Na > K	Rationed
	No. 4 (a)	Survival	34	non-infected control	0	K > Na	Rationed
	No. 4 (b)	Larval weight, bacterial load	30	non-infected control	0	K > Na	Rationed
	No. 5 (a)	Survival	37	DAT606(CC3)	1.7×10^7	Na > K	Rationed
	No. 5 (b)	Larval weight, bacterial load	30	DAT606(CC3)	1.4×10^7	Na > K	Rationed
	No. 6 (a)	Survival	36	DAT606(CC3)	2.1×10^7	K > Na	Rationed
	No. 6 (b)	Larval weight, bacterial load	30	DAT606(CC3)	1.3×10^7	K > Na	Rationed
	No. 7 (a)	Survival	72	non-infected control	0	K > Na	Rationed
	No. 7 (b)	Larval weight, bacterial load	30	non-infected control	0	K > Na	Rationed
	No. 8 (a)	Survival	78	DAT606(CC3)	1.3×10^7	K > Na	Rationed
	No. 8 (b)	Larval weight, bacterial load	30	DAT606(CC3)	1.8×10^7	K > Na	Rationed
III	No. 9 (a)	Survival	72	non-infected control	0	K > Na	Rationed
	No. 10 (a)	Survival	30	DAT606(CC3)	6.0×10^5	K > Na	Rationed
	No. 11 (a)	Survival	30	DAT561(CC12)	6.7×10^5	K > Na	Rationed
	No. 12 (a)	Survival	56	DAT585(CC13)	3.6×10^6	K > Na	Rationed
	No. 13 (a)	Survival	35	non-infected control	0	K > Na	Rationed
	No. 13 (b)	Larval weight, bacterial load	25	non-infected control	0	K > Na	Rationed
	No. 14 (a)	Survival	35	DAT606(CC3)	5.7×10^5	K > Na	Rationed
	No. 14 (b)	Larval weight, bacterial load	31	DAT606(CC3)	5.7×10^5	K > Na	Rationed
	No. 15 (a)	Survival	35	DAT561(CC12)	2.8×10^5	K > Na	Rationed
	No. 15 (b)	Larval weight, bacterial load	30	DAT561(CC12)	2.8×10^5	K > Na	Rationed
	No. 16 (a)	Survival	35	DAT585(CC13)	2.5×10^5	K > Na	Rationed
	No. 16 (b)	Larval weight, bacterial load	31	DAT585(CC13)	2.5×10^5	K > Na	Rationed

*Group-(a) larvae were used to determine mortality, and group-(b) larvae were used to measure larval weight and/or bacterial load in the larvae.

**Daily rations of the artificial diet are described in Table 3.

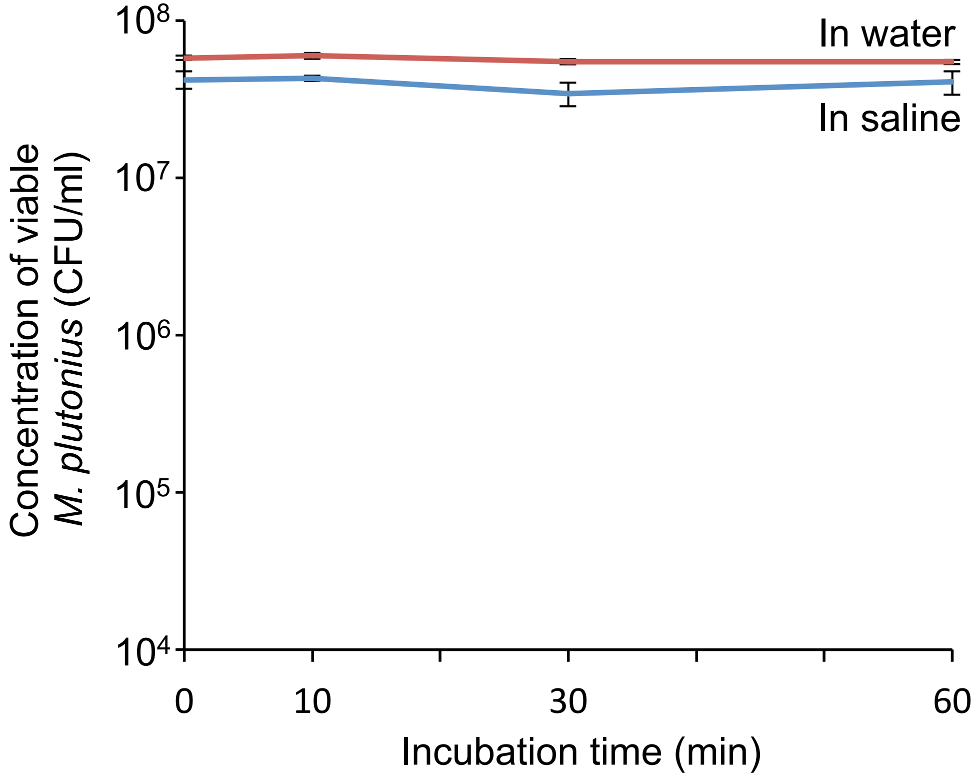

Supplementary Figure S1. Survival of *M. plutonius* DAT606(CC3) in water (red) and saline (blue). DAT606(CC3) was suspended in sterile water or saline and incubated for 10, 30 and 60 min at room temperature (25ºC). The survival of bacteria in the suspensions was investigated as described in Supplementary Methods. Data were collected from five independent suspensions and expressed as CFU/ml (mean ± SEM). There was no significant difference in the concentration of viable bacteria by the sampling period of 0, 10, 30 and 60 min (repeated ANOVA, P=0.17 and 0.47 for water and saline, respectively).

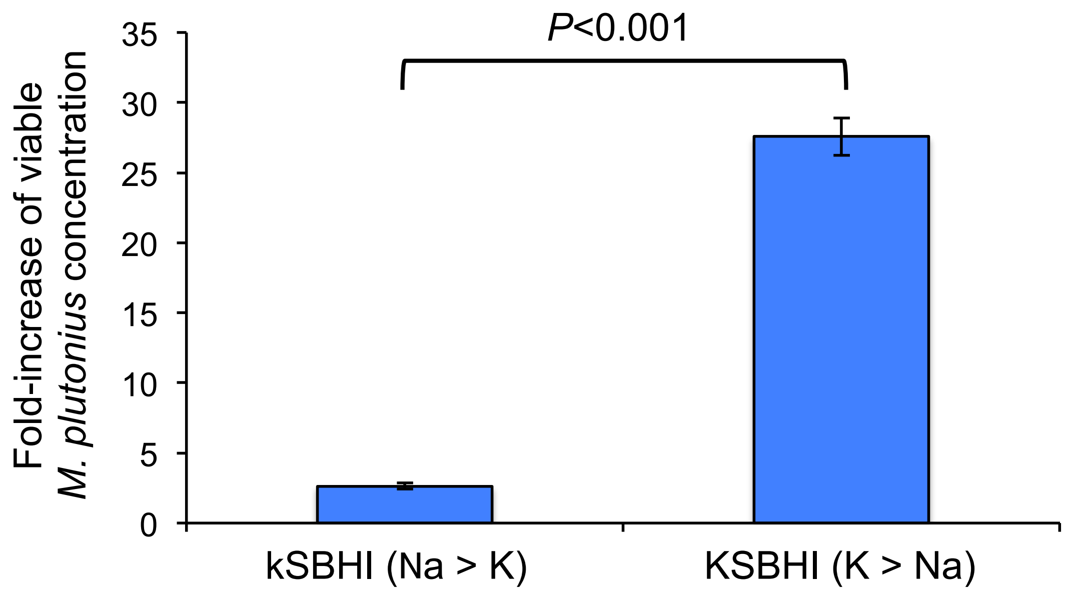

Supplementary Figure S2. Growth of *M. plutonius* DAT606(CC3) in Na-rich and K-rich liquid media. DAT606(CC3) was cultured in kSBHI broth (BHI broth with 1% soluble starch and 13.5 mM KH_2PO_4, Na/K = 3.31) and KSBHI broth (BHI broth with 1% soluble starch and 150 mM KH_2PO_4, Na/K = 0.68) for 24 h at 34ºC ± 0.5ºC under anaerobic conditions. The growth of bacteria was investigated as described in Supplementary Methods. Data were collected from five independent cultures, and the results were expressed as fold-increase relative to the concentration of viable bacteria at 0 h (mean ± SEM). The difference in the increase of DAT606(CC3) concentration between the two culture media was compared by the two-tailed Welch's *t*-test.

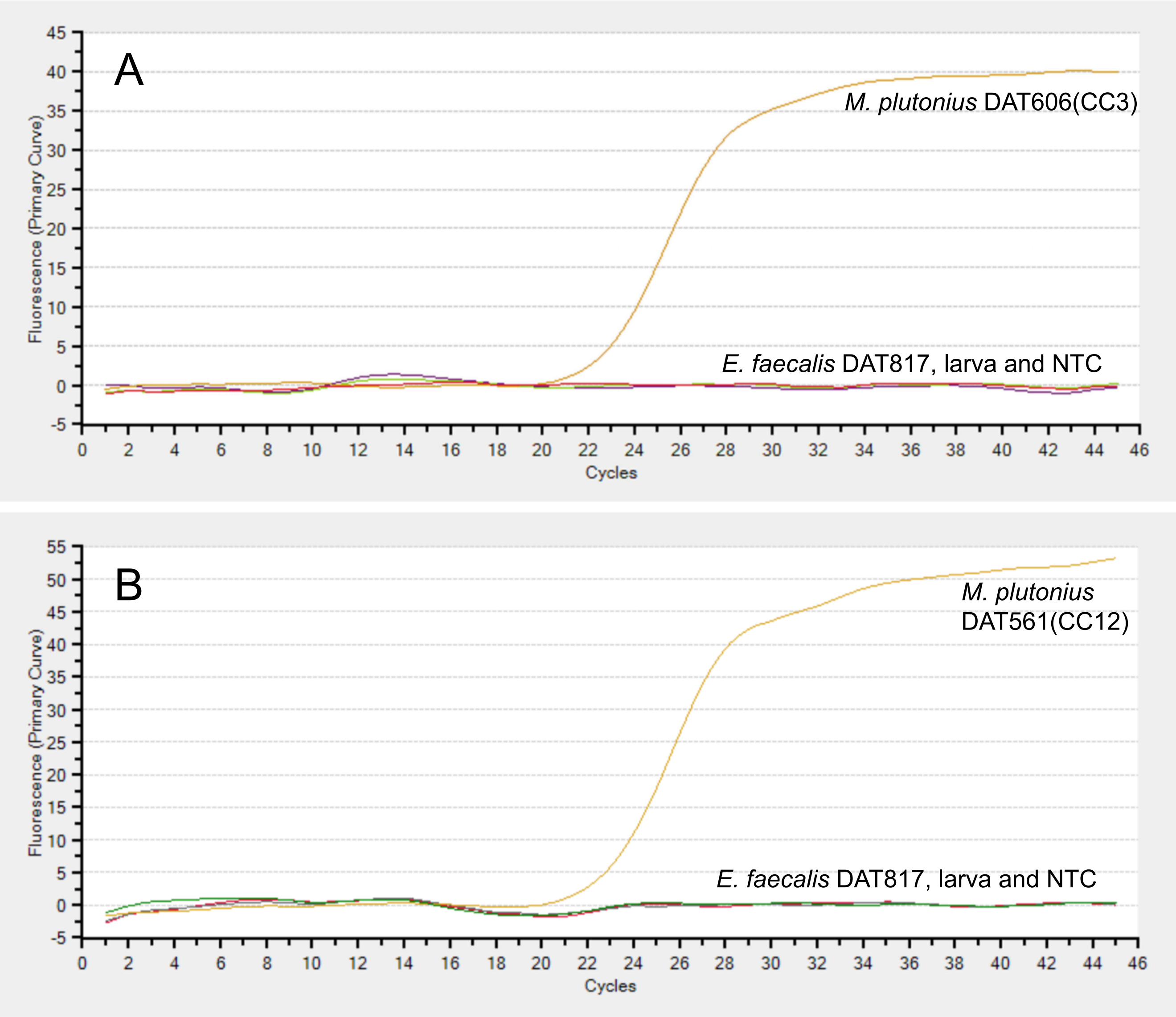

Supplementary Figure S3. Real-time PCR analysis of DNA from *M. plutonius* DAT606(CC3) (A), DAT561(CC12) (B), *E. faecalis* DAT817 (A and B) and a healthy honey bee larva (A and B). *E. faecalis* DAT817 was isolated from a honeybee larva. Primer combinations Mp-Trt-F plus Mp-Trt-R and Mp-Art-F plus Mp-Art-R were used for reactions shown in A and B, respectively. Fifty ng of DNA was used for each reaction. *M. plutonius* yielded a signal by both primer combinations, whereas no signals were recorded for *E. faecalis* DAT817, the honeybee larvae and no template control (NTC, pure water).

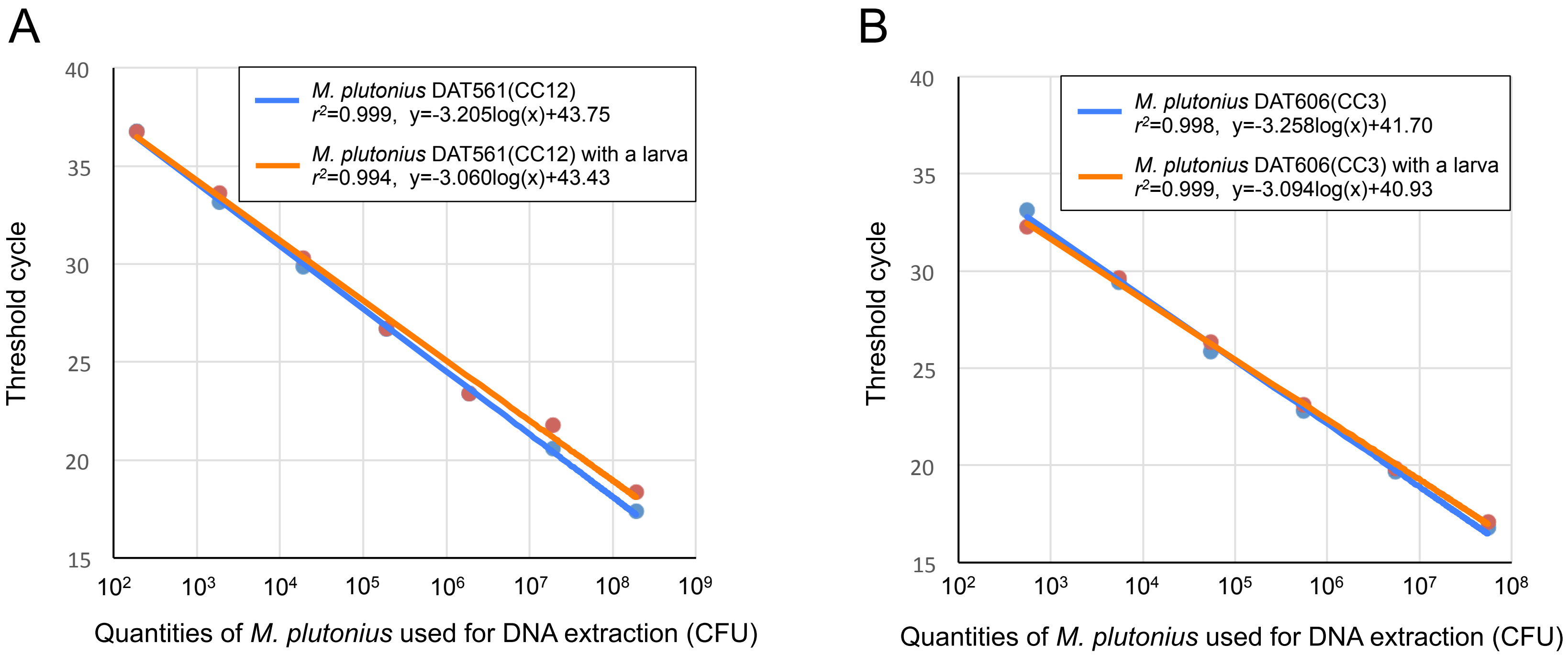

Supplementary Figure S4. Examples of real-time RT-PCR standard curve. Standard curves for the Fur family transcriptional regulator gene (MPD5_RS04175) of *M. plutonius* DAT561(CC12) (A) and the Na^{+}/H^{+} antiporter gene (MPTP_RS01890) of *M. plutonius* DAT606(CC3) (B). A series of 10-fold dilutions was made from a known concentration of *M. plutonius* suspension. *M. plutonius* DNA was extracted by DNeasy Blood & Tissue kit (Qiagen) from each dilution. To investigate the influence of larval tissue and DNA on extraction of *M. plutonius* DNA and PCR amplification efficiency, *M. plutonius* DNA was also extracted by the kit after addition of a healthy larva to each dilution. The x-axis represents quantities of *M. plutonius* used for DNA extraction. DNA was eluted with 200 μl of the elution buffer in the kit, and 1 μl of the extracted DNA was used for each reaction. The presence of the larval tissue and DNA did not affect extraction of *M. plutonius* DNA or amplification of the target genes.
